# Cervical canal stenosis due to cervical spondylotic myelopathy C4-C5: A case report

**DOI:** 10.1016/j.ijscr.2019.05.038

**Published:** 2019-06-04

**Authors:** Ahmad Jabir Rahyussalim, Ifran Saleh, Muhammad Triadi Wijaya, Tri Kurniawati

**Affiliations:** a Department of Orthopaedic and Traumatology, Faculty of Medicine, Universitas of Indonesia-Cipto Mangunkusumo General Hospital, Jakarta 10430, Indonesia; b Stem Cell and Tissue Engineering Cluster, IMERI Faculty of Medicine, Universitas Indonesia, Jakarta 10430, Indonesia; c Stem Cell Medical Technology Integrated Service Unit, Cipto Mangunkusumo General Hospital, Jakarta 10430, Indonesia

**Keywords:** Cervical spondylotic myelopathy, Decompression, Open-door laminoplasty, Neck pain, JOA, Spine

## Abstract

**Introduction:**

Cervical spondylotic myelopathy (CSM) is a complex disease that presents with various signs and symptoms of cervical spinal cord impairment that may lead to significant clinical morbidity.

**Presentation of case:**

We present the case of a 50-year old man who was diagnosed with CSM. The patient underwent decompression and posterior stabilisation with open-door laminoplasty. At the 2-month follow-up, the pain subsided, function improved significantly, and weakness disappeared. The patient was also able to defecate and urinate normally.

**Discussion:**

Cervical spondylotic myelopathy is a complex disease that may lead to significant clinical morbidity. The management requires an extensive knowledge of the anatomy, biomechanics, and surgical options. The variable clinical findings, radiological evidence and scoring system, such as JOA, are important for preoperative evaluation and individualising surgical planning. The choice of the most appropriate technique is affected by patient's clinical condition and radiologic findings as well as surgeon's experience. It is demonstrated that the Kurokawa-type laminoplasty that involves splitting the spinous processes in the midline offers the advantage of reduced bleeding as the lateral epidural venous plexus is not disturbed in comparison to that with the former Hirabayashi's expansive open-door laminoplasty. Moreover, the body symmetry is preserved; therefore, this procedure may be considered more anatomical and physiological. However, differences in the outcomes between the two approaches remain unknown.

**Conclusions:**

These findings suggest that the decompression and posterior stabilisation method may help achieve good patient outcomes.

## Highlights

• Mini plate can be used as alternative material for laminoplasty procedures.
• Modified spacer for laminoplasty is needed in case of the conventional one is not available.
• Posterior cervical laminoplasty decompression is important issue to investigate.

## 1 Introduction

Cervical spondylotic myelopathy (CSM) encompasses a wide range of symptoms and signs, including motoric, sensory and autonomous symptoms related to compression and dysfunction of the spinal cord. If treated early, this disease has a good prognosis. However, wide variations in the clinical presentation often make it difficult to establish the diagnosis. Cervical spine plays a huge role in protecting the most mobile and fragile segment of the spinal cord. The most common cervical spinal impairment is caused by degenerative changes in the surrounding tissues, resulting in spinal cord compression. One important factor is a congenitally small spinal canal that predisposes to cervical canal stenosis and subsequent myelopathy. Numerous techniques are available for treating CSM, including the anterior approach, either anterior cervical discectomy and fusion or corpectomy and strut grafting, or the posterior approach, either laminectomy or laminoplasty. Each technique has its own post-operative advantages and risks.

In this report, we present the case of a 51-year-old male patient with cervical canal stenosis due to cervical spondylotic myelopathy in C4-C5. This work has been reported in line with the SCARE criteria [1]. The patient underwent decompression and posterior stabilisation with open-door laminoplasty.

## 2 Case illustration

A 50-year-old man visited our hospital with pain in his neck that had lasted for 6 months. The patient felt pain with paraesthesia in both his shoulders that radiated to his fingers. The pain occurred intermittently and mostly during activities. There was no prior history of trauma. For 5 months, the pain and paraesthesia worsened, and he complained of weakness in both lower extremities. The patient then sought medical advice from a neurosurgeon who said that there was nerve entrapment and advised him to undergo laminoplasty. At that time, he refused to undergo surgery because he was unable to make a decision. For 4 months, the patient underwent physiotherapy; however, there was no improvement. The patient subsequently visited our hospital where he was advised to undergo laminoplasty. He worked as a contractor and mostly sat behind a desk. He had no history of diabetes or hypertension. He denied any decrease in body weight or appetite, and there was no history of chronic cough.

**Fig. 1:**
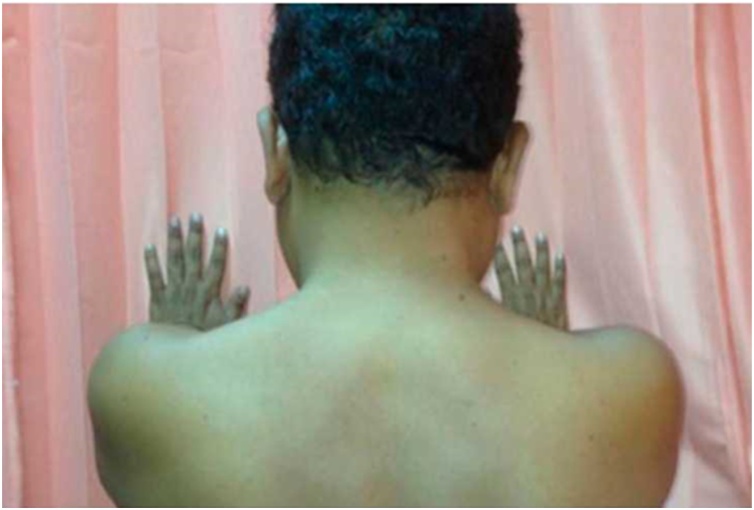
Physical examination of the cervical spine prior to surgery.

On physical examination, his general condition was unremarkable (Fig. 1). There was no tenderness on palpation along the midline. Examination of the cervical spine showed positive L'hermitte test, finger-escape test, grip-and-release test, Hoffman-Trommer sign and Spurling sign. The patient was able to move his neck normally. Further examination revealed diminished motoric strength in all extremities with positive Babinski reflex and clonus with normal patellar and Achilles tendon reflexes. He had urinary retention and faecal incontinence.

**Fig. 2:**
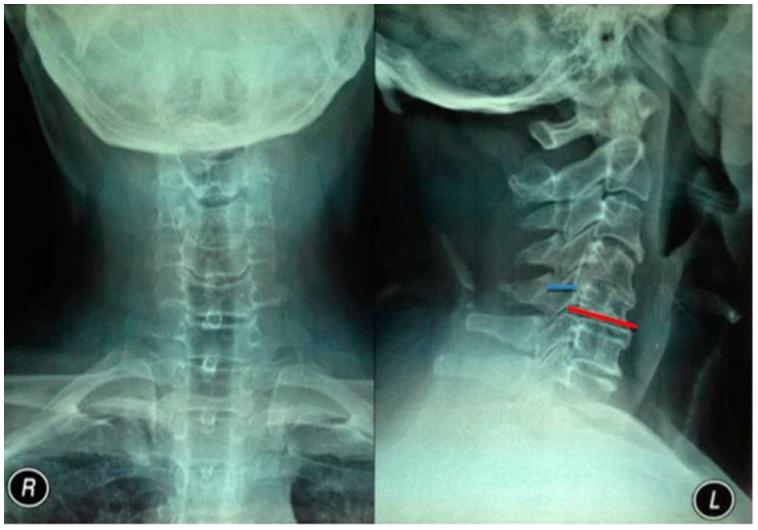
Preoperative cervical spine radiography image from (A) the anteroposterior view and (B) the lateral view.

**Fig. 3:**
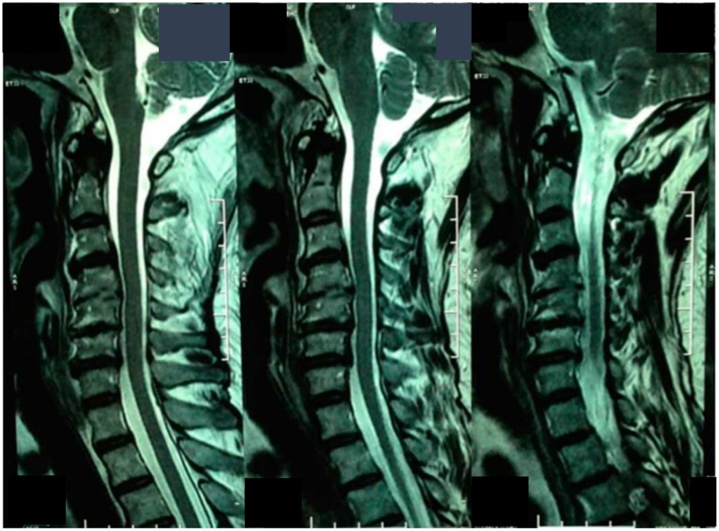
Sagittal magnetic resonance imaging showing narrowing of the spinal canal.

The patient underwent radiographic examination that showed straight cervical with mid-sagittal diameter of 10 mm and a Torg ratio of 0.37 (Fig. 2). There was osteophyte, endplate irregularity and disc-space narrowing at C4-C5 levels with spur formation at C3 through C5. Magnetic resonance imaging (MRI) examination showed cervical canal stenosis at C4-C5 levels and spinal cord compression (Fig. 3). Laboratory findings were unremarkable except for leukocytosis.

The patient was diagnosed with cervical canal stenosis due to cervical spondylotic myelopathy at C4-C5 vertebrae, with a Japanese Orthopaedic Association (JOA) Score of 11. He underwent decompression and posterior stabilisation with open-door laminoplasty under general anaesthesia. Two months after the operation, the patient felt no pain in his neck or fingers. He was able to function normally, and the weakness disappeared. The patient was able to defecate and urinate normally. The JOA score after surgery improved to 17.

## 3 Mini plate and screw placement in open-door laminoplasty

**Fig. 4:**
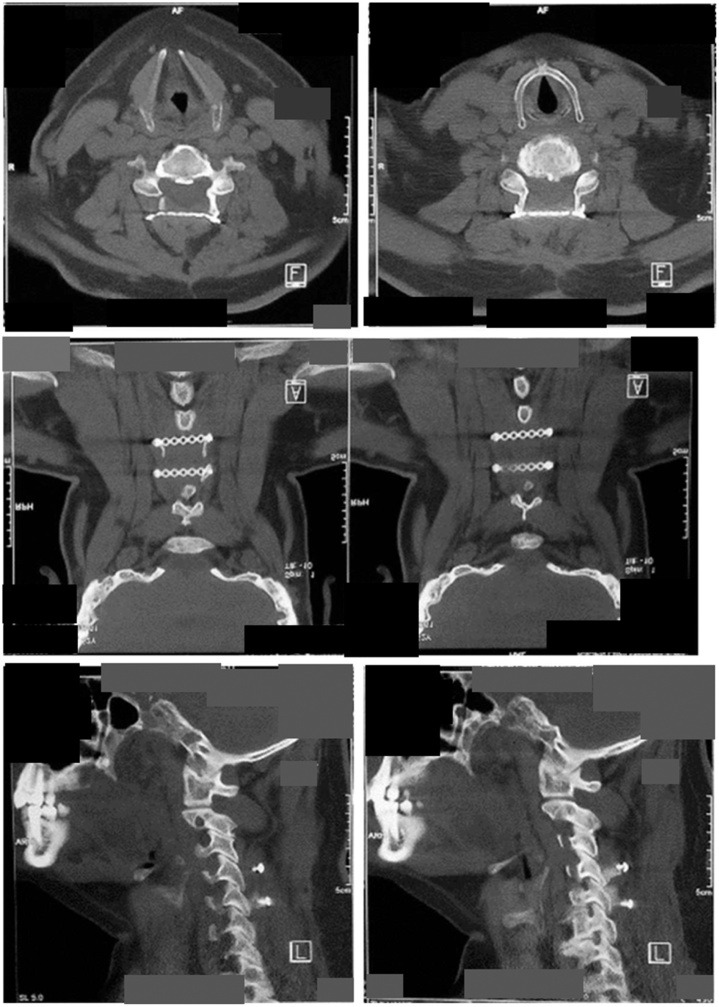
Post-operative cervical spine computed tomography scan.

Open-door laminoplasty was performed with the posterior approach of the cervical spine. The affected site was exposed, and osteotomies were made at C4-C5 on both sides. The vertebrae foramen was confirmed to be opened wider, and a mini plate was put on that level and fixated into both the pedicles (Fig. 4).

## 4 Discussion

CSM is a spectrum of disease that encompasses various signs and symptoms of cervical spinal cord impairment. The onset can be insidious with sudden worsening, with or without a history of trauma. Rao et al., reported that 5% of CSM cases had rapid onset followed by a long quiescent period, 20% showed steady but gradual progression of symptoms and signs, and 75% showed stepwise deterioration in clinical function with intervening variable periods of quiescent disease [2].

There are no pathognomonic signs or symptoms of CSM. In a review, the most common clinical presentation was gait disturbance, followed by neck pain, and referred pain to the shoulder or subscapular area. The patient may also experience clumsiness or diffuse numbness in the hands, resulting in poor fine motor skills and difficulties in holding or grasping [3].

A recent study describes the following possible sympathetic symptoms of cervical spondylosis as a result of stimulation or compression of the sympathetic nervous system due to cervical spondylopathy: vertigo, headache, tinnitus, nausea and vomiting, heart throb, hypomnesia and gastroenterologic discomfort [4].

In our case, the patient came to the hospital with symptoms related to motoric, sensory and autonomic functions of the cervical spine. The patient may be unable to perform the grip-and-release test, i.e. make a fist and rapidly release it 20 times in 10 s.

Most provocative tests are related to identify radiculopathy, spinal cord pathology, or brachial plexus pathology. Positive test result is reproduction of the radicular symptoms distant from the neck. L'hermitte's Sign is considered positive when electric-like sensations down the spine or the extremities present during passive anterior cervical flexion. Hoffman's Sign positive is flexion adduction of the ipsilateral thumb and index finger when applying passive snapping flexion of the middle finger distal phalanx.

In our case, the physical examination revealed heightened physiological reflexes, spasticity, presence of Hoffman and Babinsky pathological reflexes, clonus and positive L’hermitte sign. These signs predominate below the level of spinal cord compression.

Wada et al., used a combination of neurological symptoms and preoperative functional scoring using the JOA system and radiographic findings to determine the need for operative treatment. The most commonly observed myelopathic symptoms in the series were clumsiness of the hands, unsteady gait and numbness in the extremities. Operative treatment was recommended when these symptoms were present in combination with a JOA score <13 points and spinal cord compression in imaging studies [2].

Our patient was diagnosed with cervical canal stenosis due to cervical spondylotic myelopathy at C4-C5 vertebrae with a JOA score of 11. The JOA score along with the clinical and radiological evidence made the patient a good candidate for operative intervention [2]. Early surgery is essential for interfering with the natural history of the disease. There is strong evidence that prognosis is markedly improved when the interval between the onset of symptoms and surgery is less than a year [3].

The patient underwent decompression and posterior stabilisation with open-door laminoplasty under general anaesthesia. Posterior approaches may be considered when the pathology is located at the posterior portion of the spinal canal. When compared to anterior approaches, posterior procedures offer several advantages for CSM treatment. Some of these factors are that they may not require fusion of that vertebral level, and it enables direct visualisation of the spinal canal and wide decompression of the spinal cord and the nerve roots [3,5].

Laminectomy had been extensively used for treating cervical myelopathy caused by multisegmental spondylosis, ossification of the posterior longitudinal ligament, and development of spinal canal stenosis. Laminectomy provided unsatisfactory clinical results because of the intraoperative spinal cord injury, post-operative progression of cervical kyphosis, and worsening of neurological functions in relation to the formation of scar tissue over the dural sac (laminectomy membrane); therefore, cervical laminoplasty was developed to overcome the drawbacks of laminectomy. There are several objectives of performing cervical laminoplasty, such as the follows: (1) to achieve adequate multilevel spinal cord decompression with expansion of the spinal canal, (2) to prevent the formation of post-operative severe scar over the dural sac, (3) to avoid destabilisation of the posterior structures of the spine and (4) to preserve physiological mobility of the cervical spine [8].

Various laminoplasty techniques have been described. All these variations are designed to reposition the laminae and expand the spinal canal while retaining the dorsal elements to protect the dura from scar formation and to preserve post-operative cervical stability and alignment [7]. A recent study, has demonstrated that there is no evidence of the superiority of laminectomy with fusion over laminoplasty in reducing neck pain in CSM patients [3,5,6].

In Hirabayashi's expansive open-door laminoplasty, the spinal cord is decompressed asymmetrically because the door opens on one side and hinges on the other side. In contrast, Kurokawa's double-door laminoplasty, described by Kurokawa in 1982, expands the canal symmetrically as the opening is created in the midline.

One of the advantages of the double-door technique is that the decompression occurs directly posterior to the cord, resulting in less bleeding from the lateral epidural veins that often accompany the open-door technique. Adhesions between the dura and ventral side of the lamina are freed. The laminar opening can be fixed with a suture passing through the facet capsules and the lamina. In the initial description of the double-door procedure, the canal was left open. However, several techniques have been proposed to span the space between the gapped lamina and to protect the spinal cord. These include the use of ceramic/hydroxyapatite spacers, iliac crest bone graft, rib autograft, or, as described by Kurokawa, resected spinous process autograft fixed between the lamina with wires [7].

In our case, a plate for bony-bridge instead of bone block was used. A new titanium plating design was developed for easier fixation. This is comparable to a study by Rhee et al., wherein the new titanium plate design was used without graft and showed a union rate of 77% at 6 months. Our union rate can also be compared to a study by Tanaka et al., that evaluated bone healing in 88 patients who underwent open-door laminoplasty with hydroxyapatite spacers and autogenous graft spacers with union rates on the hinged side of 84% and 79%, respectively, at 6 months.

The primary disadvantage of the double-door technique is that it could be technically challenging. This technique also potentially puts the spinal cord more at risk than the open-door technique because the dura is just deep to the spinous process that is split with a burr or saw. Foraminotomy is also technically demanding and may cause disruption of the hinge [7].

Most studies report outcomes using the JOA scoring system, documenting the mean pre- and post-operative scores and the rate of recovery. In the literature, recovery rates of at least 50%–70% following laminoplasty have been consistently reported although rates as high as 90% have also been reported. Multiple authors have verified the reliable outcomes of laminoplasty in the short to midterm; in contrast, few series have shown improvement in the neurologic status following laminoplasty that is maintained in the long-term. However, differences in outcomes between the two approaches remain unknown. Several systematic reviews and meta-analysis suggest that cervical laminoplasty approach is not superior to the other based on the post-operative radiological data and complication rate, although open-door laminoplasty had higher post-operative JOA scores than French-door laminoplasty [7,9].

## 5 Conclusion

Cervical spondylotic myelopathy is a complex disease that may lead to significant clinical morbidity. The management requires an extensive knowledge of the anatomy, biomechanics, and surgical options. The variable clinical findings, radiological evidence and scoring system, such as JOA, are important for preoperative evaluation and individualising surgical planning. The choice of the most appropriate technique is affected by patient's clinical condition and radiologic findings as well as surgeon's experience. It is demonstrated that the Kurokawa-type laminoplasty that involves splitting the spinous processes in the midline offers the advantage of reduced bleeding as the lateral epidural venous plexus is not disturbed in comparison to that with the former Hirabayashi's expansive open-door laminoplasty. Moreover, the body symmetry is preserved; therefore, this procedure may be considered more anatomical and physiological. However, differences in the outcomes between the two approaches remain unknown.

## Conflicts of interest

We disclosed any financial and personal relationships with other people or organisations that could inappropriately influence (bias) our work.

## Sources of funding

There is nothing to declare.

## Ethical approval

No need ethical approval because this is a case report which was only observational.

## Consent

Written informed consent was obtained from the patient for publication of this case report and accompanying images. A copy of the written consent is available for review by the Editor-in-Chief of this journal on request.

## Author contribution

Ahmad Jabir Rahyussalim, Muhammad Triadi Wijaya: study concept or design,

Tri Kurniawati: data collection, writing the paper.

Ifran Saleh:data analysis or interpretation.

## Registration of research studies

It was not registered.

## Guarantor

Ahmad Jabir Rahyusalim.

## Provenance and peer review

Not commissioned, externally peer-reviewed.
